# Nonlinear Microscale Mechanics of Actin Networks Governed by Coupling of Filament Crosslinking and Stabilization

**DOI:** 10.3390/polym14224980

**Published:** 2022-11-17

**Authors:** Mike E. Dwyer, Rae M. Robertson-Anderson, Bekele J. Gurmessa

**Affiliations:** 1 Department of Physics and Astronomy, Bucknell University, Lewisburg, PA 17837, USA; 2 Department of Physics and Biophysics, University of San Diego, San Diego, CA 92110, USA

**Keywords:** microrheology, α-actinin, actin, crosslinking, optical tweezers, phalloidin, nonlinear viscoelasticity, bundling

## Abstract

Actin plays a vital role in maintaining the stability and rigidity of biological cells while allowing for cell motility and shape change. The semiflexible nature of actin filaments—along with the myriad actin-binding proteins (ABPs) that serve to crosslink, bundle, and stabilize filaments—are central to this multifunctionality. The effect of ABPs on the structural and mechanical properties of actin networks has been the topic of fervent investigation over the past few decades. Yet, the combined impact of filament stabilization, stiffening and crosslinking via ABPs on the mechanical response of actin networks has yet to be explored. Here, we perform optical tweezers microrheology measurements to characterize the nonlinear force response and relaxation dynamics of actin networks in the presence of varying concentrations of α-actinin, which transiently crosslinks actin filaments, and phalloidin, which stabilizes filamentous actin and increases its persistence length. We show that crosslinking and stabilization can act both synergistically and antagonistically to tune the network resistance to nonlinear straining. For example, phalloidin stabilization leads to enhanced elastic response and reduced dissipation at large strains and timescales, while the initial microscale force response is reduced compared to networks without phalloidin. Moreover, we find that stabilization switches this initial response from that of stress stiffening to softening despite the increased filament stiffness that phalloidin confers. Finally, we show that both crosslinking and stabilization are necessary to elicit these emergent features, while the effect of stabilization on networks without crosslinkers is much more subdued. We suggest that these intriguing mechanical properties arise from the competition and cooperation between filament connectivity, bundling, and rigidification, shedding light on how ABPs with distinct roles can act in concert to mediate diverse mechanical properties of the cytoskeleton and bio-inspired polymeric materials.

## 1. Introduction

The mechanical properties of networks of actin filaments have been the topic of fervent investigation for decades due to the key roles they play in maintaining the stability and rigidity of biological cells while also allowing for cell motility and shape change [1,2,3,4,5]. This multifunctionality is enabled by the semiflexible nature of actin filaments and the myriad actin-binding proteins (ABP) that can, e.g., crosslink, stabilize, stiffen, and bundle actin filaments, resulting in viscoelastic networks with wide-ranging mechanical properties [2,6,7,8,9,10,11]. A notable feature of these networks, relevant to both cellular biology and polymer physics, is their unique nonlinear response to strain and the roles that filament stiffness, crosslinking, and concentration play in this signature nonlinear response [12,13,14,15].

In the absence of ABPs, actin filaments above a critical concentration form entangled networks in vitro with linear viscoelastic properties that can be described reasonably well by reptation-based models for entangled semiflexible polymers [16,17]. The nonlinear response of entangled actin networks is more complex and has been shown to exhibit varying degrees of stress stiffening and softening depending on the spatiotemporal scale of the strain and the network concentration [12,13,18,19,20]. Forced disentanglement, shear-thinning, entropic stretching, and strain alignment have also all been implicated as important features of the nonlinear response of entangled actin networks [12,13,21,22].

Introducing crosslinking ABPs into entangled actin networks greatly enhances the elastic contribution to the mechanical response by suppressing thermal fluctuations and disentanglement. However, dissipative processes still contribute appreciably to the mechanics at low ABP:actin ratios *R* and for transient crosslinkers such as α-actinin, which continuously bind and unbind to actin filaments to allow for network rearrangement [23]. Even in the case of static crosslinking, such as in studies that use avidin proteins to bind biotinylated actin filaments, dissipation, relaxation, and plastic rearrangement have been reported to contribute to the response to nonlinear straining due to forced rupturing of crosslinker bonds [12,24,25,26]. The type and concentration of crosslinking ABPs also control the extent to which ABPs isotropically crosslink actin filaments, to form a homogeneous well-connected network of individual filaments, or bundle filaments, to form a more loosely connected and heterogeneous network of stiff fibers (i.e., multi-filament bundles). These alterations to network connectivity and fiber stiffness play important roles in the diverse mechanical and structural features that have been reported for actin networks crosslinked by a range of ABPs including, e.g., α-actinin, scruin, fascin, and filamin [13,14,27,28,29,30,31,32,33,34,35,36,37,38].

While bundling via ABPs increases the stiffness of actin network fibers [31,36,39,40,41,42,43,44], stabilizing ABPs such as phalloidin stiffen individual filaments [45,46,47,48]. Phalloidin selectively binds to filamentous actin to suppress treadmilling [46,49], thereby accelerating the polymerization rate and lowering the critical polymerization concentration [46,49]. Phalloidin-stabilized filaments have been reported to have a persistence length of $l_{p}≃$ 17 μm, nearly twice $l_{p}≃$ 10 μm measured for unstabilized actin filaments [48,50,51,52]. Moreover, stabilized filaments have been reported to form bundles similar to those formed by high concentrations of crosslinking ABPs [35]. Filament bundling contributes to the viscoelastic response of actin networks in a manner highly dependent on network concentration due to competing effects of increased filament rigidity and reduced network connectivity [7,18,35,53,54,55,56].

**Figure 1:**
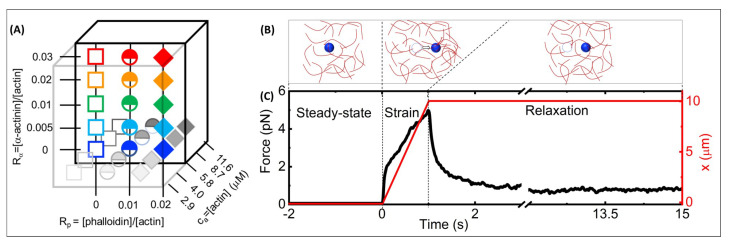
**Optical tweezers microrheology characterizes the nonlinear force response of actin networks with varying molar concentrations of actin monomers, α-actinin and phalloidin**: (**A**) Cartoon of experimental parameter space of filamentous actin networks with varying: α-actinin:actin molar ratios, $R_{\alpha }$ = 0–0.03 (*y*-axis), phalloidin:actin molar ratios $R_{p}$ = 0–0.02 (*x*-axis), and molar actin monomer concentrations, $c_{a}$ = 2.9–11.6 μm (*z*-axis). (**B**) Optical tweezers microrheology (OTM) measurement protocol: an optically trapped 4.2-μm diameter microsphere probe (blue) embedded in an actin network (red) is: held fixed for 15 s to allow the network and probe to reach steady-state and establish a baseline (left, steady-state), displaced *x* = 10 μm at a constant speed *v* = 10 μm/s by moving the piezoelectric stage relative to the trap (middle, strain), and held fixed while the network relaxes to a new steady-state (right, relaxation). The dashed circles represent the initial probe position, and the arrow indicates the trap motion during strain. (**C**) A sample OTM measurement showing the measured force exerted on the trapped probe (black) and the stage position versus acquisition time. The dashed vertical lines divide the data into the three phases depicted in (**B**).

Here, we investigate the coupled roles of stabilization, crosslinking, and entanglement density on the microscale nonlinear response of in vitro actin networks. Specifically, we use optical tweezers microrheology to measure the nonlinear force response and relaxation dynamics of entangled networks of actin filaments with actin monomer concentrations of $c_{a}=2.9–11.6$ μm, [phalloidin]:$c_{a}$ ratios of $R_{p}=0–0.02$, and [α-actinin]:$c_{a}$ ratios of $R_{\alpha }=0–0.03$ (Figure 1). We find that phalloidin stabilization increases the elastic response and stiffness that crosslinked networks exhibit at mesoscopic scales. However, at the microscale, stabilization surprisingly suppresses stress stiffening and lowers the force response. We also uncover intriguing nonmonotonic dependence of stiffness and relaxation dynamics on $R_{p}$, $R_{\alpha }$, and $c_{a}$, which we rationalize as arising from the distinct roles that filament bundling and connectivity play in the nonlinear response of actin networks.

## 2. Materials and Methods

**Proteins:** Rabbit skeletal actin, α-actinin, and Acti-Stain 555 Phalloidin (Cytoskeleton, Inc, Denver, CO, USA) were reconstituted to 46 μM, 10 μm and 14 μm and stored, respectively, at −80 ${}^{{}^{\circ}}C$ in G-buffer [2 mM Tris pH 8.0, 0.5 mM DTT, 0.1 mM ${\mathrm{CaCl}}_{2}$, 0.2 mM ATP], at −80 ${}^{{}^{\circ}}C$ in [4 mM Tris-HCl pH 7.6, 4 mM NaCl, 20 μm EDTA, 1% (*w*/*v*) sucrose, 0.2% (*w*/*v*) dextran], and at −20 ${}^{{}^{\circ}}C$ in 100% methanol. Alexa-568 labeled actin (Thermo Fisher Scientific) was reconstituted to 35 μm and stored at −80 ${}^{{}^{\circ}}C$ in G-buffer [2 mM Tris pH 8.0, 0.5 mM DTT, 0.1 mM ${\mathrm{CaCl}}_{2}$].

**Network Formation:** We mixed unlabeled G-actin and either (i) 568-actin or (ii) 555-phalloidin with oxygen scavenging agents (4.5 μg/mL glucose, 0.005% β-mercaptoethanol, 4.3 μg/mL glucose oxidase, 0.7 μg/mL catalase) to final actin concentrations of 2.9–11.6 μm in F-buffer [10 mM Imidazole pH 7.0, 50 mM KCl, 1 mM ${\mathrm{MgCl}}_{2}$, 1 mM EGTA, 0.2 mM ATP]. We included 568-actin or 555-phalloidin at (i) a 1:10 ratio of labeled:unlabeled actin or (ii) phalloidin:actin ratios of $R_{p}$ = 0.01 and 0.02. We also included α-actinin at molar α-actinin:actin ratios of $R_{\alpha }$ = 0, 0.005, 0.01, 0.02, and 0.03 (Figure 1A), which have been reported to result in isotropic, crosslinked networks with small-scale bundles forming above $R_{\alpha }≃$ 0.02 [23,35].

We immediately flowed the solution into a 3 mm × 0.5 mm × 0.1 mm sample chamber comprising double-sided tape as a spacer between a glass slide and coverslip, sealed the chamber with epoxy, and allowed the actin monomers to polymerize into filaments at room temperature for 1 h. For all networks, we added a trace of 4.2-μm diameter polystyrene microspheres (Bangs Laboratories, Inc, Fishers, IN, USA) prior to polymerization to allow for microrheology measurements (Figure 1B). The predicted mesh sizes of the filamentous actin networks at the actin mass concentrations we examined span ζ = 0.3/$\sqrt{C_{a}}$ = 0.42–0.85 μm, where $C_{a}$ is the actin mass concentration in mg/mL [57,58].

**Microscopy:** To characterize the structure of the different actin networks, we used a Leica TCS SP8 laser scanning confocal microscope with a 63 × 1.4 NA objective and 561 nm laser to image fluorescently labeled filaments in actin networks. We collected *z*-stacks of 21 images of 512 × 512 pixels, each with a *z*-depth of 0.5 μm. The resulting *z*-stacks are 123 × 123 × 10.5 μm. Based on our previous studies [20,28], we expect the network structure we capture from confocal imaging to be similar to the network structure in the vicinity of the microsphere probes used in our microrheology measurements (described below), thereby allowing us to relate structural characteristics of the network to the nonlinear force response we measure.

**Microrheology:** We used an optical trap built around an IX73 fluorescence microscope (Olympus, Melville, NY, USA) with a 1064 nm Nd:YAG fiber laser (BKtel, RPMC Lasers, Inc, O Fallon, MO, USA) focused with a 60× 1.42 NA oil immersion objective (UPLXAPO60XO, Olympus). A position-sensing detector (PSM2-10Q PSD/OT-301, On-Trak Photonics, Inc, Irvin, CA, USA) measured the deflection of the trapping laser, which is proportional to the force acting on the trapped probe over our entire force range. The trap stiffness was calibrated via Stokes drag in water [59] and passive equipartition methods [60]. During measurements, a probe embedded in the network is trapped and moved 10 μm in the +x-direction, relative to the camera field of view, at a constant speed of *v* = 10 μm/s relative to the sample chamber via steering of a nanopositioning piezoelectric stage (PDQ-250, Mad City Laboratories, Madison, WI, USA). The strain speed and distance were chosen to be large compared to the characteristic length and timescales of the networks to ensure we are probing the force response in the nonlinear regime, in which the networks are pushed far from equilibrium. We previously showed that the critical strain speed for the onset of nonlinearity in entangled actin networks is ∼3 μm/s, [19], so we chose a speed of 10 μm/s to be well above this speed, but slow enough that we could reliably move the particle the full 10 μm distance without the bead being forced out of the trap by the resistive force of the network. This speed and distance were also similar to the ones used in [18,28] to measure the nonlinear force response of entangled and statically crosslinked actin networks.

**Figure 2:**
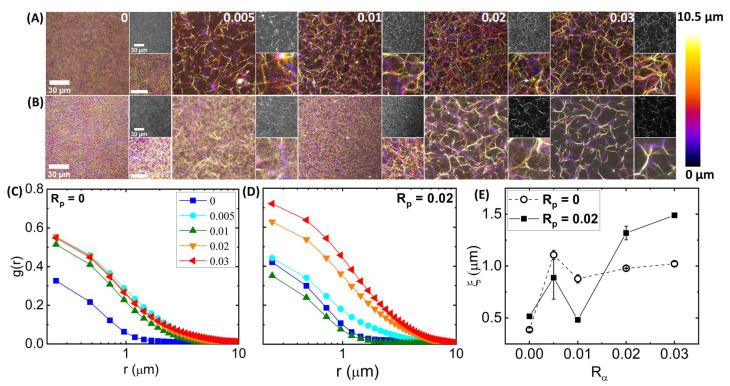
**The structure and mobility of actin networks depend on the degree of filament crosslinking and stabilization**. (**A**,**B**) Laser scanning confocal microscopy imaging of 5.8 μm fluorescence-labeled actin networks with varying α-actinin:actin molar ratios $R_{\alpha }$ = 0–0.03, increasing from left to right as indicated at the top right corner of each image. Networks (**A**) without phalloidin ($R_{p}$ = 0) and (**B**) with a phalloidin:actin molar ratio $R_{p}$ = 0.02 depict the effect of filament stabilization on network structure. For each [$R_{\alpha }$,$R_{p}$] combination, three-dimensional stacks of 21 images, each of 0.5 μm thickness, were captured using a Leica TCS SP8 laser scanning confocal microscope with 60 × 1.4 NA objective. Large left-hand images are color-coded *z*-projections of the image stacks, with the color scale shown to the right and scale bars representing 30 μm. Small images are single slices from the stack (**top**) and zoom-ins of the *z*-projections (**bottom**) with a scale bar indicating 10 μm. (**C**,**D**) Spatial image autocorrelation functions, g(r) versus radial distance *r*, computed from each image of each *z*-stack shown in (**A**,**B**), quantify the average feature size of images. Each g(r) curve shown is the average over those obtained from each of the 21 images of the corresponding *z*-stack shown in (**A**) for each $R_{\alpha }$, as denoted in the legend, for networks with (**C**) $R_{p}$ = 0 and (**D**) $R_{p}$ = 0.02. (**E**) The correlation lengths ξ as a function of $R_{\alpha }$, obtained by fitting each average g(r) curve shown in (**C**) (open symbols, dashed connecting lines) and (**D**) (closed symbols, solid connecting lines) to an exponentially decaying function: g(r)=g(0)exp(−r/ξ). Error bars are obtained by fitting g(r) for each image of the corresponding *z*-stack and computing the standard error across the 21 independent ξ values measured at different *z*-heights in the image stack.

We measured both the laser deflection and stage position at a rate of 20 kHz during the three phases of each trial: equilibration (5 s), strain (1 s), and relaxation (15 s) (Figure 1B). We found that force data from measurements performed in the −x-direction are statistically indistinguishable from those in the +x-direction (data not shown). We did not perform measurements in the *y*-direction, which corresponds to the shorter dimension of our sample chamber (∼0.5 mm), but the isotropic structure of the networks we investigated (see Figure 2) suggests that the force response is likewise isotropic.

**Figure 3:**
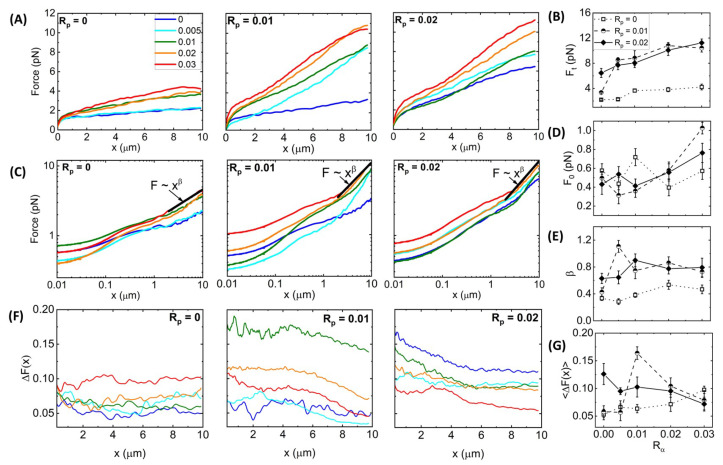
**Synergistic effects of crosslinking and stabilization enable sustained elastic response of actin networks to nonlinear straining**. (**A**) Force F(x) versus stage position *x* measured for actin networks subject to nonlinear straining (see Figure 1). Different curves in each panel correspond to α-actinin:actin molar ratios of $R_{\alpha }$ = 0–0.03, color-coded according to the legend in (**A**). Different panels display data for phalloidin:actin molar ratios of $R_{p}$ = 0 (**left**), $R_{p}$ = 0.01 (**middle**), and $R_{p}$ = 0.02 (**right**). (**B**) Terminal force reached at the end of the strain $F_{t}$ as a function of $R_{\alpha }$ for $R_{p}$ = 0 (open squares, dotted connecting lines), $R_{p}$ = 0.01 (half-filled circles, dashed connecting lines), and $R_{p}$ = 0.02 (solid diamonds, solid connecting lines). (**C**) Data shown in (**A**) plotted on a log–log scale to highlight the trends seen for the initial force $F\left(x=0\right)=F_{0}$ and power-law scaling of F(x) near the end of the strain (x≈1). Fitting the large strain data to a power-law F(x)∼$x^{\beta }$ yields the scaling exponent β that quantifies the degree of elastic storage maintained at the end of the strain. (**D**) Initial force measured at the beginning of the strain $F_{0}$ and (**E**) power-law scaling exponent β determined from fits to F(x)∼$x^{\beta }$ depicted in (**B**) versus $R_{\alpha }$ for $R_{p}$ = 0 (open squares, dotted connecting lines), $R_{p}$ = 0.01 (half-filled circles, dashed connecting lines), and $R_{p}$ = 0.02 (solid diamonds, solid connecting lines. (**F**) Fractional spread in force ΔF(x) for each position *x* and each condition, determined by computing the standard error across 30 individual trials and normalizing by the average value plotted in (**A**): ΔF(x) = $SE_{F}/\langle F\left(x\right)\rangle$. (**G**) ΔF(x) averaged over the strain position *x*, resulting in a single value for each curve shown in (**F**) with error bars denoting the standard error across *x* values. Error bars shown in (**B**,**D**,**E**) represent the standard error of values computed from the 30 individual trials that comprise each average force curve shown in (**A**).

**Figure 4:**
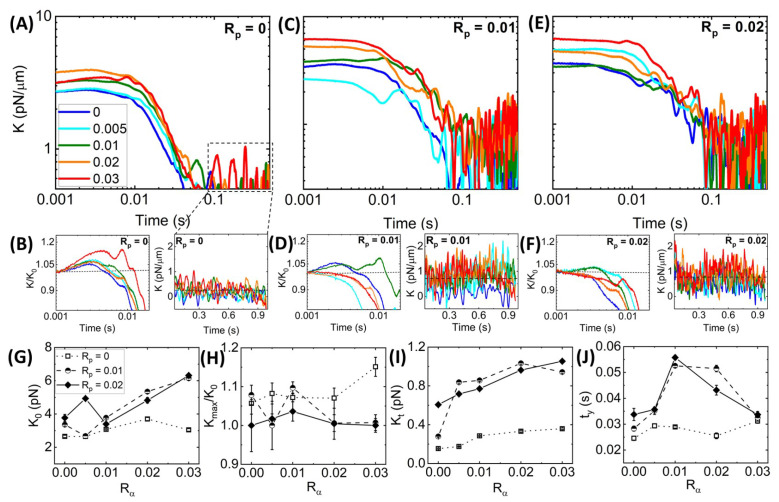
**Stabilization suppresses the stress-stiffening behavior of crosslinked networks while increasing the response stiffness and associated yield times**. (**A**) Effective differential modulus K(t) = dF(x,t)/dx as a function of time during strain t=v/x, computed for each average force curve shown in Figure 3A. The color-coded curves are for $R_{\alpha }$ values indicated in the legend. (**B**) K(t) data shown in (**A**) normalized by the corresponding initial value $K_{0}$ (**left**) with the horizontal dashed line at $K\left(t\right)/K_{0}$ = 1 guiding the eye to show stress stiffening ($K\left(t\right)/K_{0}>1$) or softening ($K\left(t\right)/K_{0}<1$). Zoom-ins of K(t) near the end of the strain (**right**) where K(t) is approximately constant, with the dashed horizontal line denoting the average. (**C**–**F**) Metrics plotted in (**A**,**B**) for networks with $R_{p}$ = 0.01 (**C**,**D**) and $R_{p}$ = 0.02 (**E**,**F**). (**G**–**J**) Quantities computed from the data shown in (**A**–**F**) as functions of $R_{\alpha }$ for $R_{p}$ = 0 (open squares, dotted connecting lines), $R_{p}$ = 0.01 (half-filled circles, dashed connecting lines), and $R_{p}$ = 0.02 (solid diamonds, solid connecting lines): (**G**) Initial differential modulus $K_{0}$; (**H**) Degree of stress stiffening, quantified as $K_{max}/K_{0}$ and equal to 1 for networks which only display softening (i.e., $K_{max}$ = $K_{0}$); (**I**) Terminal stiffness $K_{t}$ computed by averaging over the K(t) data shown in the right-hand panels of (**B**,**D**,**F**); (**J**) Yield time $t_{y}$, defined as the time at which K(t) = $K_{0}/e$. Error bars shown in (**G**–**J**) represent the standard error of values computed from 5 random subsets of the data, as described in Materials and Methods.

**Figure 5:**
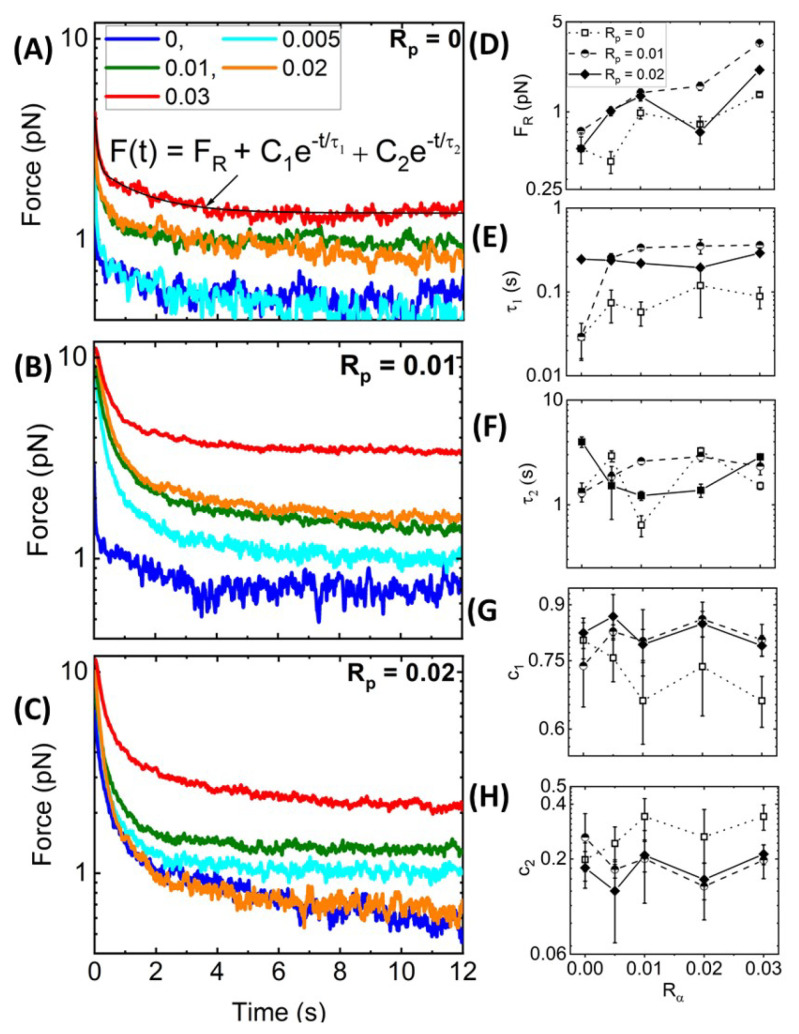
**Strain-induced force exponentially relaxes over time to a residual value $F_{r}$ dependent on $R_{p}$ and $R_{\alpha }$**. (**A**–**C**) Time-dependent relaxation of force F(t) following strain for actin networks with varying $R_{\alpha }$ values, color-coded according to the legend, and with $R_{p}$ values of (**A**) 0, (**B**) 0.01, and (**C**) 0.02. All F(t) curves are well-described by a sum of two exponential decays with a long-time residual $F_{R}$: $F\left(t\right)=F_{R}+C_{1}\exp\left(-t/\tau_{1}\right)+C_{2}\exp\left(-t/\tau_{2}\right)$ as indicated by the representative fit (solid black line) shown in (**A**). (**D**–**H**) The residual force $F_{R}$ (**D**), decay times $\tau_{1}$ (**E**) and $\tau_{2}$ (**F**), and corresponding fractional coefficients, $c_{1}$ = $C_{1}/\left(C_{1}+C_{2}\right)$ (**G**) and $c_{2}$ = $C_{2}/\left(C_{1}+C_{2}\right)$ (**H**), are determined from the fits and plotted as functions of $R_{\alpha }$ for $R_{p}$ = 0 (open squares, dotted connecting lines), $R_{p}$ = 0.01 (half-filled circles, dashed connecting lines), and $R_{p}$ = 0.02 (solid diamonds, solid connecting lines). Error bars shown in (**D**–**H**) represent the standard error of values computed from 5 random subsets of the data as described in Materials and Methods.

**Figure 6:**
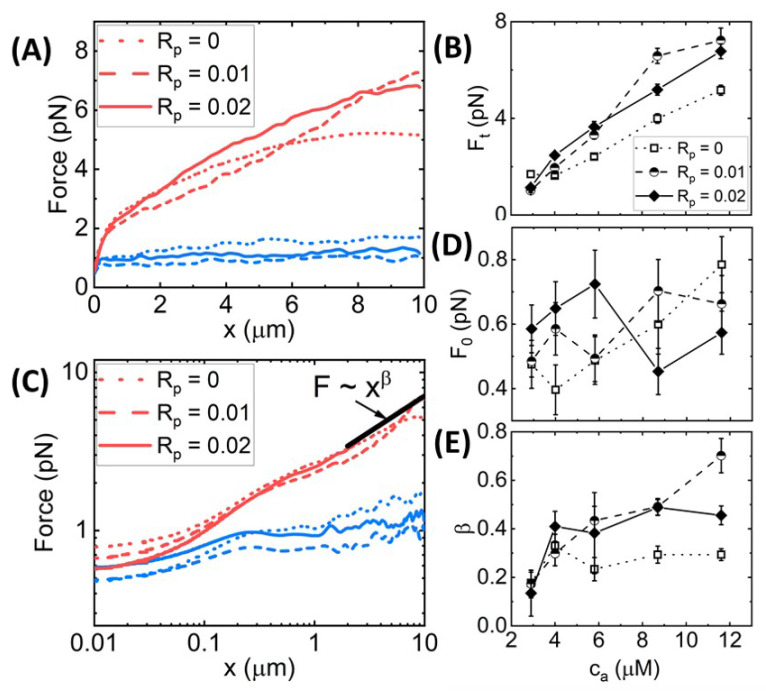
**Effect of phalloidin stabilization and actin concentration on the nonlinear force response of entangled actin networks**. (**A**) Force F(x) versus stage position *x* for actin networks subject to nonlinear straining (see Figure 1), for actin concentrations of $c_{a}$ = 2.9 μm (blue) and $c_{a}$ = 11.6 μm (red) and phalloidin:actin ratios of $R_{p}$ = 0 (dotted lines), $R_{p}$ = 0.01 (dashed lines), and $R_{p}$ = 0.02 (solid lines). (**B**) Terminal force reached at the end of the strain $F_{t}$ as a function of $c_{a}$ for $R_{p}$ = 0 (open squares, dotted connecting lines), $R_{p}$ = 0.01 (half-filled circles, dashed connecting lines), and $R_{p}$ = 0.02 (solid diamonds, solid connecting lines). (**C**–**E**) Data shown in (**A**) plotted on a log–log to highlight the trends seen for the initial force $F\left(x=0\right)=F_{0}$ (plotted in (**D**)) and power-law scaling of F(x) near the end of the strain (plotted in (**E**)). Error bars shown in (**B**,**D**,**E**) represent the standard error of values computed from the 30 individual trials that comprise each average force curve shown in (**A**,**C**) and SI Figure S2.

Each force curve shown in Figure 3A,C, Figure 5A–C and Figure 6A,C is from a different sample and is an average of 30 trials performed using 30 different probes at different locations, separated by >100 μm, throughout the sample chamber. The error bars shown in Figure 3B,D,E, Figure 5D and Figure 6B,D,E are the standard error of values computed from each of the 30 individual force curves shown in SI Figures S1 and S2. The error bars shown in Figure 4G–J and Figure 5E–H, which plot metrics that are inherently more susceptible to noise present in individual trials, are the standard error of values computed from 5 random subsets of 6 trials each. Measurements repeated on a second independent sample for each condition showed statistically indistinguishable trends (SI Figure S6), demonstrating the reproducibility and validity of our results. Custom-written LabVIEW code was used to perform measurements and acquire data, while data analysis was performed with custom-written Matlab programs.

## 3. Results and Discussion

We first aim to characterize the effect of phalloidin stabilization on the nonlinear force response, relaxation dynamics and steady-state structure of actin networks crosslinked with α-actinin.

**Network structure.** To verify that the actin networks we examined follow the previously reported structural trends [35], and to shed light on the structural impact of phalloidin stabilization, we acquired three-dimensional fluorescence confocal image stacks of labeled actin networks. Figure 2A,B, which shows *z*-projections of images, color-coded by *z*-height, and single images and zoom-ins to the right of each colorized projection, displays network structure without (Figure 2A, $R_{p}$ = 0) and with (Figure 2B, $R_{p}$ = 0.02) phalloidin for increasing crosslinker concentrations $R_{\alpha }$ (left to right). Without phalloidin, there is a stark difference between networks with and without crosslinkers, with the unlinked network ($R_{\alpha }$ = 0) appearing much more homogeneous and densely filled with individual filaments (Figure 2A). The lack of clear discernible filaments indicates enhanced Brownian noise compared to the crosslinked cases, as filaments can fluctuate over the course of the acquisition. Conversely, the crisp, high-contrast fibers seen in crosslinked networks (Figure 2B), coupled with larger visible dark regions void of filaments, indicate that the crosslinked fibers are highly rigid and static over the course of acquisition and that the fibers are likely small bundles rather than single filaments.

To quantify these structural variations, we computed the spatial image autocorrelation function g(r), where *r* is the radial distance between two pixels for each image (shown in greyscale in Figure 2) in the image stack. The autocorrelation g(r) is a measure of how correlated the intensities of two pixels a given distance *r* away from one another are. The distance *r* over which g(r) decays, often termed the correlation length ξ, indicates the average size of features in the image. For an isotropic fiber network, ξ is comparable to the mesh size. As shown in Figure 2C, the autocorrelation is lower for all *r* values for the unlinked network, indicating minimal spatial structure and increased thermal noise compared to the crosslinked networks. Introducing crosslinkers significantly increases the structural correlation for all radial distances and also increases the length scale over which g(r) decays, both trends indicative of larger and more pronounced structural features. By fitting each autocorrelation function to an exponential decay function, g(r)=g(0)exp(−r/ξ), we extracted the correlation length, ξ, which corroborates our qualitative description. Namely, ξ increases ∼2-fold upon crosslinking, but it remains roughly constant for all $R_{\alpha }>$ 0 values (Figure 2E).

Interestingly, the images shown in Figure 2B suggest that adding phalloidin to the networks shown in Figure 2A may either inhibit *or* promote the restructuring that α-actinin crosslinking induces, depending on $R_{\alpha }$. Specifically, for $R_{\alpha }$≤ 0.01, phalloidin stabilization appears to have little impact on the apparent density or homogeneity of the networks, as seen by the similar g(r) curves and ξ values for $R_{\alpha }$ = 0, 0.005 and 0.01 (Figure 2D,E). In contrast, in the absence of phalloidin, there is evident small-scale bundling for $R_{\alpha }$ = 0.005 and 0.01 networks, as shown by the larger g(r) and ξ values compared to the $R_{\alpha }$ = 0 network (Figure 2C,E). On the other hand, for $R_{\alpha }$ > 0.01, phalloidin stabilization seems to increase α-actinin mediated bundling, as indicated by the thicker and brighter fibers and larger empty voids in the last two columns of Figure 2B compared to Figure 2A. This increased bundling is evidenced in the larger g(r) values in Figure 2D compared to Figure 2C for $R_{\alpha }$ > 0.01 (i.e., red and orange curves), and the larger ξ values of phalloidin-stabilized ($R_{p}$ = 0.02) versus nonstabilized ($R_{p}$ = 0) networks for $R_{\alpha }$ > 0.01 in Figure 2E.

**Nonlinear Stress Response.** To determine how the seemingly antagonistic and synergistic effects of stabilization and crosslinking, suggested in Figure 2, impact the nonlinear force response of the networks, we performed optical tweezers microrheology measurements described in Methods and Figure 1. In brief, we pull an optically trapped probe through a distance *x* = 10 μm at a constant speed of *v* = 10 μm/s through the network and measure the force the network exerts to resist the strain. Following the strain, we hold the probe fixed at the final strain position and measure how the force decays over time. For reference, in a purely elastic material, the force F(x) would increase linearly with strain distance *x*, with the slope dF/dx indicating the effective spring constant or stiffness, while a viscous fluid would nearly instantly reach an *x*-independent plateau. Following the strain, an elastic material would retain the induced force indefinitely, while, for a viscous fluid, the force would drop to zero immediately upon halting the probe motion. Entangled and crosslinked actin networks have been shown to exhibit viscoelastic features intermediate to these extremes, depending on the actin and crosslinker concentration [18,28,53,54].

Figure 3A shows average force response curves for networks of varying $R_{\alpha }$ values, with each panel showing networks with a fixed phalloidin:actin ratio: $R_{p}$ = 0 (left), $R_{p}$ = 0.01 (middle), and $R_{p}$ = 0.02 (right). As shown, the force F(x) generally increases with increasing $R_{\alpha }$ for all cases; however, the functional dependence on $R_{\alpha }$ is distinct for different phalloidin concentrations. Moreover, phalloidin stabilization substantially increases the resistive force compared to $R_{p}$ = 0 for all crosslinker ratios. We quantify these trends by examining the terminal force $F_{t}$ reached at the end of the strain as a function of $R_{\alpha }$ (Figure 3B). We observe that without phalloidin, $F_{t}$ increases ∼2-fold at $R_{\alpha }$ = 0.01, but it remains nearly constant as $R_{\alpha }$ is increased further, similar to the insensitivity of the structure to $R_{\alpha }$ (Figure 2). Conversely, for $R_{\alpha }$ = 0.02, the terminal force, which is substantially larger than $F_{t}$ for $R_{\alpha }$ = 0, increases monotonically with $R_{\alpha }$. Intriguingly, the reduced stabilization case ($R_{p}$ = 0.01) exhibits a non-monotonic dependence of $F_{t}$ on $R_{\alpha }$, transitioning from a value close that for $R_{p}$ = 0 in the absence of crosslinking to values that are higher than the $R_{p}$ = 0.02 network for $R_{\alpha }$ = 0.005–0.02, after which $F_{t}$ drops modestly.

To shed light on the mechanisms underlying these complex trends, we note that the functional forms of the F(x) curves differ for different crosslinker and phalloidin concentrations, such that the initial force $F_{0}$ may exhibit different dependence on $R_{\alpha }$ and $R_{p}$ than the terminal force $F_{t}$. To better visualize the initial force response and the dependence of *F* on *x*, we show the data from Figure 3A on a log–log scale (Figure 3C), from which we observe that, indeed, the small strain response displays a more complex dependence on $R_{\alpha }$ and $R_{p}$, as quantified in Figure 3D. Without crosslinkers (i.e., $R_{\alpha }$ = 0), phalloidin stabilization actually reduces $F_{0}$, whereas for $R_{p}$ > 0.01, stabilization increases $F_{0}$ (Figure 3D). A similar phenomenon has been reported for entangled composites of actin filaments and rigid microtubules, whereby composites with more microtubules compared to actin exhibited lower $F_{0}$ values compared to actin-rich composites, but this trend flipped at larger length scales where the resistive force was substantially higher for microtubule-rich composites [61]. This work showed that the increased initial force for actin-rich composites arose from the decreased mesh size and increased flexibility compared to microtubule-rich composites, which, in turn, increased the poroelastic contributions to the initial stress response [61]. Conversely, the lower terminal force $F_{t}$ arose from the semiflexible actin filaments being able to more readily dissipate induced stress compared to their rigid microtubule counterparts. Similarly, we may understand the reduced initial force and increased terminal force for phalloidin-stabilized filaments as arising from stiffening and bundling of filaments, making them more akin to microtubules, compared to actin networks without phalloidin.

Figure 3C also reveals a discrete shift at x≃ 1 μm to a steeper power-law dependence of the force on strain distance, $F\left(x\right)∼x^{\beta }$, which is more pronounced for phalloidin-stabilized networks. In other words, rather than softening to a viscous-dominant regime in which β∼0, as has been previously reported for both entangled and crosslinked actin networks [46,47,48], phalloidin-stabilized networks appear to stiffen at large strains, reaching scaling close to the elastic limit β∼1 (Figure 3E). Moreover, the scaling exponent displays a nonmonotonic dependence on crosslinking density, with the most pronounced stiffening occurring for 0 < $R_{\alpha }$ < 0.03.

Our results suggest that both fiber stiffness and mesh size play important roles in the force response, which we also expect to affect the heterogeneity between trials measured in different regions of the sample and across different samples. As the probe diameter of *a* = 4.2 μm is an order of magnitude larger than the predicted mesh size (ζ≃ 0.6 μm) and the measured correlation length ξ of the entangled actin network, the probe senses an effectively homogeneous continuum as it moves through the [$R_{\alpha }$ = 0,$R_{p}$ = 0] network. However, if the mesh size increases significantly due to bundling, as indicated in Figure 2 for networks with high $R_{\alpha }$ and $R_{p}$ > 0, then the probe may detect these structural heterogeneities as it moves through the network. Moreover, while the semiflexibility of individual actin filaments allows them to bend, stretch, and reorient during the strain such that the bead does not encounter discrete rigid entities, this assumption does not necessarily hold for networks of rigid fibers [61] or highly crosslinked polymers [62,63].

To quantify this heterogeneity, we computed the fractional spread in force values ΔF(x) = $SE_{f}/F\left(x\right)$ that contribute to each F(x) curve shown in Figure 3A,B, where $SE_{f}$ is the standard error across the 30 individual force values f(x) measured at each *x* for each condition (Figure 3F). As shown, without phalloidin stabilization, ΔF(x) is largely independent of strain distance and unaffected by $R_{\alpha }$ until $R_{\alpha }>$ 0.01 in which ΔF(x) increases modestly. Phalloidin stabilization leads to generally larger fractional spreads than without stabilization ($R_{p}$ = 0) for all crosslinker ratios and decreases with increasing strain distance. These general trends can be seen in Figure 3G, which also shows that the *x*-averaged fractional force spread 〈ΔF(x)〉 displays an emergent nonmonotonic dependence on $R_{\alpha }$, with a maximum at $R_{\alpha }$ = 0.005 for the more weakly stabilized networks ($R_{p}$ = 0.01). The *x*-dependence suggests that the network is heterogeneous on microscales that tend toward homogeneity at scales larger than ∼4 μm. Substantial bundling and increased stiffness of fibers, without strong connectivity between fibers, may contribute to this effect, which we explore further below.

**Time-varying Network Stiffness during Strain.**Figure 3 shows that all networks exhibit varying degrees of elastic stiffness and viscous dissipation that depend not only on the network composition (i.e., $R_{\alpha }$ and $R_{p}$), but also on the strain distance *x*. To quantify these nonlinear stress characteristics, we evaluate the effective differential modulus K=dF/dx, which quantifies the elasticity or stiffness of the network as a function of time t=x/v during the strain, with a purely viscous response yielding K≈ 0 (Figure 4).

Previous studies have shown that statically crosslinked actin networks subject to nonlinear straining exhibited initial stress stiffening, i.e., increasing *K*, followed by softening (decreasing *K*) to an *x*-independent regime [24,28,64]. Without phalloidin stabilization (Figure 4A), we see similar behavior, with all networks exhibiting initial stiffness $K_{0}$ and stress stiffening $K\left(t\right)/K_{0}>0$ that is generally more pronounced at higher $R_{\alpha }$ values (Figure 4B, left). After stiffening to a maximum value $K_{max}$, networks soften to a nearly viscous terminal regime with small stiffness values $K_{t}$ that are largely independent of *x* (Figure 4B, right).

Adding phalloidin to the networks increases the initial and terminal stiffness values, $K_{0}$ and $K_{t}$ (Figure 4C–F), as we might expect given the increased elastic contributions to the force response seen in Figure 3 and the increased filament rigidity that phalloidin confers. However, quite unexpectedly, phalloidin stabilization suppresses the stress-stiffening behaviors seen in Figure 4B, such that the majority of networks for both phalloidin concentrations exhibit purely softening behavior (Figure 4D,F). Moreover, this suppression is stronger for higher $R_{\alpha }$ values and for the higher phalloidin concentration $R_{p}$ = 0.02. Stress stiffening is typically associated with affine stretching and alignment with the strain, as opposed to non-affine bending and dissipative fluctuations, which are most prevalent in the response of crosslinked networks and entangled networks subject to nonlinear straining [18,28,61]. As such, the softening of phalloidin-stabilized networks, most pronounced at higher $R_{\alpha }$ values, suggests enhanced bundling as compared to networks with lower $R_{\alpha }$ values and no stabilization, as seen in Figure 2, which comes at the cost of strong network connectivity necessary for affine stretching and deformation.

These complex effects can be seen more clearly in Figure 4G–J in which the initial stiffness $K_{0}$, the relative stiffening $K_{max}/K_{0}$, and the terminal stiffness $K_{t}$ for all three $R_{p}$ values are plotted versus $R_{\alpha }$. We find that $K_{0}$ is not only larger for phalloidin-stabilized networks compared to the $R_{p}$ = 0 case, but the increase with increasing crosslinking is substantially steeper. Conversely, we see nearly the opposite trend for the relative stiffening in which the $R_{p}$ = 0 network generally has the highest degree of stiffening, whereas most of the values for the stabilized networks exhibit the floor value of $K_{max}/K_{0}$ = 1, indicative of softening. Despite the lack of stiffening, phalloidin-stabilized networks retain substantially more elastic stiffness in the steady-state (*t*-independent) region of the stress response compared to the non-stabilized network. Taken together, our results suggest that the increased rigidity of the phalloidin-stabilized bundles causes an immediate elastic response as their rigid-rod-like conformation inhibits entropic stretching (required for stiffening) and dissipative bending and fluctuations (required for $K_{t}\to 0$).

We expect the competition between rigidification and connectivity to non-trivially impact the timescales required for the network to yield or relax to a steady-state stiffness, which we quantify as the yield time $t_{y}$ at which *K* reaches *K* = $K_{0}/e$. Figure 4J reveals that without stabilization, $t_{y}$ is largely unaffected by the degree of crosslinking, whereas both stabilized networks exhibit a stark nonmonotonic dependence on $R_{\alpha }$, with the maximum yield time reached at $R_{\alpha }$ = 0.01. Moreover, $t_{y}$ values for stabilized networks are nearly identical to the nonstabilized network at the highest crosslinker density ($R_{\alpha }$ = 0.03) and in the absence of crosslinking ($R_{\alpha }$ = 0). This trend suggests that the stiffness of the individual filaments may not affect the yield time as much as the propensity to crosslink and bundle. In the following section, we explore this interpretation and more closely investigate the impact of $R_{\alpha }$ and $R_{p}$ on network relaxation dynamics.

**Nonlinear Relaxation Dynamics.** As described in Figure 1B,C, following the 10 μm strain, we hold the bead fixed at this maximum strain position and measure the relaxation of the induced force over time. Recall that purely elastic systems maintain the induced force indefinitely (no relaxation), while viscous systems exhibit immediate and complete dissipation. Figure 5A–C, which displays the relaxation profiles that follow the force response curves shown in Figure 3, shows that all networks undergo some degree of relaxation while also maintaining some residual force $F_{R}$ at the end of the relaxation period. In most cases, $F_{R}$ increases with increasing $R_{\alpha }$ and $R_{p}$, as shown in Figure 5D, indicating that crosslinking and stabilization both contribute to enhancing elastic storage, with crosslinking having a more significant impact. As in previous studies on crosslinked and bundled actin networks [28,61], we find that, for all networks, the relaxation of the force to the residual plateau $F_{R}$ can be described well by a sum of two decaying exponentials with a long-time residual: $F\left(t\right)=C_{1}\exp\left(-t/\tau_{1}\right)+C_{2}\exp\left(-t/\tau_{2}\right)+F_{R}$. The two distinct characteristic decay times, $\tau_{1}$ and $\tau_{2}$, determined from the fits of the data to this function, are measures of the timescales associated with two independent relaxation mechanisms. The corresponding coefficients, $C_{1}$ and $C_{2}$, are measures of the relative contributions of each mechanism to the overall relaxation.

To understand the mechanisms underlying each exponential term, we turn to predicted relaxation timescales for entangled semiflexible polymers [65]. The fastest predicted relaxation mechanism is the mesh time $\tau_{mesh}$, i.e., the time for entangled polymers to feel the surrounding mesh [18,61,65], which depends on the network mesh size ζ and filament persistence length $l_{p}$ as $t_{mesh}$≃$4\zeta^{4}/l_{p}$ [18,65,66,67]. Assuming $\zeta ≃0.3/C_{a}^{1/2}≃0.6\;\mu$m for systems with and without phalloidin and considering the increased persistence length of phalloidin-stabilized filaments, the predicted $t_{mesh}$ values are ∼0.05 s and ∼0.03 s in the absence and presence of phalloidin, respectively. Our measured $\tau_{1}$ values for $R_{p}$ = 0 are comparable to the predicted mesh time, with an average value of $\langle \tau_{1}\rangle ≃$ (0.07 ± 0.03) s and a modest increase with increasing $R_{\alpha }$ (Figure 5E). This increase may be due to increasing mesh size as filaments begin to form bundles such that the effective concentration of fibers comprising the network is lower. Surprisingly, in contrast to predictions, the addition of phalloidin substantially increases $\tau_{1}$, with $R_{\alpha }$-averaged values of $\langle \tau_{1}\rangle ≃$ (0.27 ± 0.14) s and (0.24 ± 0.04) s for $R_{p}$ = 0.01 and 0.02, respectively. Given the strong dependence of $t_{mesh}$ on the mesh size, these longer timescales may arise from the increasing mesh size of phalloidin-stabilized networks due to bundling, which reduces the effective concentration of distinct fibers comprising the network (as we see in Figure 2). Specifically, a <2-fold increase in ζ with $R_{\alpha }$ would result in the ∼8-fold longer timescales we measure compared to theoretical predictions (i.e., ∼0.24 s versus ∼0.03 s).

Unlike our measured $\tau_{1}$ values, $\tau_{2}$ appears largely independent of both $R_{p}$ and $R_{\alpha }$ suggesting that the underlying relaxation mechanism is independent of filament stiffness, connectivity, or other properties of the network (Figure 5F). We also recall that α-actinin is a transient crosslinker, and its dissociation rate from actin has been shown to control the low-frequency relaxation of α-actinin-crosslinked actin networks in the linear regime [23]. These previous studies have reported dissociation-mediated relaxation timescales of ∼1–2.5 s, with minimal dependence on $R_{\alpha }$. Our measured $\tau_{2}$ values are in close agreement with these reported relaxation times [23] and are similarly insensitive to $R_{\alpha }$, with $\langle \tau_{2}\rangle$ = (1.94 ± 1.11) s, (2.19 ± 0.63) s, and ( 2.19 ± 1.20) s, for $R_{p}$ = 0, 0.01 and 0.02, respectively. We thus attribute our slow relaxation timescale to transient unbinding and rebinding of α-actinin crosslinks that allow for dissipative network rearrangement.

We note that our measured $\tau_{1}$ and $\tau_{2}$ values are faster than those reported for entangled and statically crosslinked actin networks, which both exhibit relaxation timescales on the order of ∼1 s and ∼10 s [18,28]. The slow relaxation timescales in the previous studies were attributed to disengagement of entangled actin filaments from their entanglement tubes. Here, the turnover rate of α-actinin is faster than this disengagement rate such that it can allow for ‘slow’ stress relaxation to occur on a faster timescale. Moreover, $\tau_{2}$ is comparable to the previously reported fast timescales (∼1 s), which were attributed to lateral hopping of entangled filaments out of their entanglement tubes due to constraint release and force-induced crosslinker unbinding [18,28], both of which are similar relaxation mechanisms to transient α-actinin crosslinking. Finally, $\tau_{1}$ values, which agree with $t_{mesh}$, are in line with the relaxation timescales measured for actin networks bundled by counterions, which likewise agree with corresponding predicted $t_{mesh}$ values [7]. This agreement suggests that this relaxation mode may play a larger role in the relaxation of bundled or otherwise stiffened filaments compared to networks of individual semiflexible filaments.

To shed further light on the impact of crosslinking and stabilization on the relaxation dynamics, we also evaluate the relative contributions of the fast and slow relaxation mechanisms (associated with $\tau_{1}$ and $\tau_{2}$, respectively) to the overall stress relaxation by evaluating the fractional coefficient of the corresponding exponential decaying term, $c_{1}$ = $C_{1}/\left(C_{1}+C_{2}\right)$ and $c_{2}$ = $C_{2}/\left(C_{1}+C_{2}\right)$. As shown in Figure 5G,H, similar to the relaxation timescales, the coefficients are largely insensitive to the crosslinker density $R_{\alpha }$. We also observe that the fast relaxation contributes more to phalloidin-stabilized networks compared to $R_{p}$ = 0 networks, as seen from the larger $c_{1}$ values in Figure 5G, with crosslinker unbinding playing a lesser role (Figure 5H). This effect likely arises from the fact that the values of $\tau_{1}$ are larger for phalloidin-stabilized networks compared to $R_{p}$ = 0 networks, such that fast relaxation dynamics span more of the measured relaxation phase, thus contributing more to the relaxation. Moreover, as crosslinking is the predominant mechanism driving the viscoelastic response of non-stabilized networks, we expect the crosslinker dynamics to play an important role in the relaxation. Conversely, for phalloidin-stabilized networks, the increased filament stiffness and bundling also contribute substantially to the viscoelasticity, such that dissipative fluctuations on the scale of the mesh size (occurring over $\tau_{mesh}≃\tau_{1}$) contribute to the dynamics more strongly.

**Dependence of Nonlinear Force Response on Actin Concentration.** The complex effects of phalloidin stabilization on the mechanics of crosslinked actin networks suggest that similar stabilization may play an important role in the nonlinear response of entangled actin networks without crosslinkers. Namely, the impacts of filament stiffening and bundling, which rigidify the network while also increasing the mesh size and altering fiber connectivity, suggest that phalloidin stabilization may depend strongly on the actin concentration, which dictates the network mesh size and degree of connectivity. To this end, we performed the same experimental protocols and analyses as described in the preceding sections for entangled actin networks ($R_{\alpha }$ = 0) at five different actin concentrations ranging from 2× lower to 2× higher than $c_{a}$ = 5.8 μm used for the crosslinked networks.

We find that the dependence of the force response on stabilization is surprisingly weak, as shown in Figure 6, in contrast to the crosslinked network results shown in Figure 2, Figure 3, Figure 4 and Figure 5. This general finding suggests that phalloidin stabilization alone, without crosslinking, is not sufficient to substantially stiffen actin networks. Rather, the synergistic coupling between crosslinking and stabilization appears to be required to confer the substantial stiffening seen in, e.g., Figure 3B,E and Figure 4G,I,J. The relative insensitivity of the nonlinear force response to stabilization in the absence of crosslinkers may also indicate that crosslinking is necessary to facilitate substantial bundling of phalloidin-stabilized filaments that would otherwise remain as an entangled network of individual filaments. We explore these results and hypotheses below.

We first evaluate the force response during strain for varying actin concentrations $c_{a}$ with $R_{p}$ = 0, 0.1, and 0.2 (Figure 6). As shown in Figure 6A,B and SI Figure S3, we find that the terminal force $F_{t}$ monotonically increases with $c_{a}$ for all networks, independent of $R_{p}$. However, phalloidin stabilization does modestly increase F(x) and its dependence on strain *x* compared to the $R_{p}$ = 0 case. Conversely, Figure 6C,D shows that the initial force $F_{0}$ is higher for phalloidin-stabilized networks at low actin concentrations ($c_{a}$ ≤ 5.8 μm) but transitions to being lower than $R_{p}$ = 0 networks at higher $c_{a}$. This result suggests that at low $c_{a}$, when the entanglement density is lower, and network connectivity is weaker, the stiffness of the filaments plays a principal role in the microscale force response. However, as the concentration, and thus the entanglement density and connectivity, increases, $F_{0}$ for the $R_{p}$ = 0 networks becomes larger, indicating that it is the network connectivity that dominates over filament stiffness. Similar to our results for crosslinked networks (Figure 3), the lower initial force for the $R_{p}$ > 0 networks suggests the presence of weak bundling at high actin concentrations that decreases connectivity compared to the $R_{p}$ = 0 case.

Furthermore, similar to crosslinked networks (Figure 3), the power-law scaling of F(x) at large length scales, i.e., $F\left(x\right)∼x^{\beta }$, is higher for phalloidin-stabilized networks compared to $R_{p}$ = 0 networks, indicating that filament stiffness plays the principal role in maintaining elastic storage at large length scales while crosslinks and entanglements can rearrange and dissipate induced stress over these spatiotemporal scales. However, while phalloidin stabilization substantially increased the heterogeneity in the force response of crosslinked networks (Figure 3, SI Figure S1), suggestive of increased bundling, we observe no similar increase for entangled networks (SI Figures S2 and S3). The phalloidin-mediated increase in $F_{t}$ and β is also weaker for entangled networks compared to crosslinked networks for all $c_{a}$ and $R_{\alpha }$ values (Figure 6B,E), in line with our understanding that bundling of phalloidin-stabilized networks is facilitated by crosslinkers that bridge bundles.

The effect of phalloidin stabilization on the stress stiffening and softening during strain, as well as the force relaxation following strain, is markedly weaker for entangled networks compared to crosslinked networks (Figure 3). While the initial and terminal stiffness $K_{0}$ and $K_{t}$ are modestly larger for phalloidin-stabilized networks, the degree of stiffening $K_{max}/K_{0}$ and the timescale over which the network yields to the terminal regime $t_{y}$ are largely insensitive to stabilization. Likewise, the relaxation timescales, $\tau_{1}$ and $\tau_{2}$, their relative contribution to the relaxation, $c_{1}$ and $c_{2}$, and the residual force $F_{R}$ are all statistically indistinguishable across varying $R_{p}$ values (SI Figure S5D–H).

## 4. Conclusions

Networks of semiflexible actin filaments play critical roles in various cellular processes ranging from cell motility to stiffening to mechanosensation. Central to these mechanical processes are actin-binding proteins that crosslink and stabilize actin filaments. The structural changes that these ABPs mediate, in turn, tune the mechanical response of the networks to strain. Here, we used optical tweezers microrheology to elucidate the coupled effects of crosslinking and stabilization on the nonlinear force response and relaxation dynamics of entangled actin networks. Specifically, we studied actin networks crosslinked by the transient crosslinker α-actinin at ABP:actin molar ratios of $R_{\alpha }$ = 0–0.03, and stabilized by phalloidin at ABP:actin molar ratios of $R_{p}$ = 0, 0.01, and 0.02. Our results reveal complex relationships between crosslinking and stabilization that lead to emergent mechanical properties such as suppressed stress stiffening and enhanced sustained elasticity. Notably, the effect of stabilization on the force response features of entangled actin networks is substantially weaker than for crosslinked networks, independent of the actin concentration. Taken together, our results demonstrate that crosslinking and phalloidin-mediated stiffening of actin filaments act synergistically to promote bundling while maintaining connectivity—both essential for soliciting a strong elastic response to nonlinear straining. However, at sufficiently high crosslinking, bundling comes at the cost of connectivity, leading to a nonmonotonic dependence on key mechanical properties such as yield time, terminal response elasticity, and microscale heterogeneity. Beyond the relevance to cytoskeletal mechanics, our results may generally provide insight into the coupled roles of filament rigidity and connectivity on the nonlinear response of polymer networks and hydrogels.

## Data Availability

The data presented in this study are available on request from the corresponding author.

## Supplementary Materials

The following supporting information can be downloaded at: https://www.mdpi.com/article/10.3390/polym14224980/s1.
