# Supplementary material for: Nonlinear Microscale Mechanics of Actin Networks Governed by Coupling of Filament Crosslinking and Stabilization
Source: Polymers (Basel). 2022 Nov 17;14(22):4980. doi: 10.3390/polym14224980 (PMC9696012; doi:10.3390/polym14224980)
Supplement: Supplementary file 1 [file polymers-14-04980-s001.zip › polymers-2022506-supplementary.pdf]

.
https://www.overleaf.com/project/63333ff2e4affe6e72637961

Article

Nonlinear microscale mechanics of actin networks governed by coupling of filament crosslinking and stabilization

Mike E. Dwyer [1], Rae M. Robertson-Anderson [2], and Bekele J. Gurmessa[1]

[1] Department of Physics and Astronomy, Bucknell University, Lewisburg, PA 17837, USA
[2] Department of Physics and Biophysics, University of San Diego, San Diego, CA 92110, USA
* Correspondence: bjg018@bucknell.edu

1. Supplemental Figures

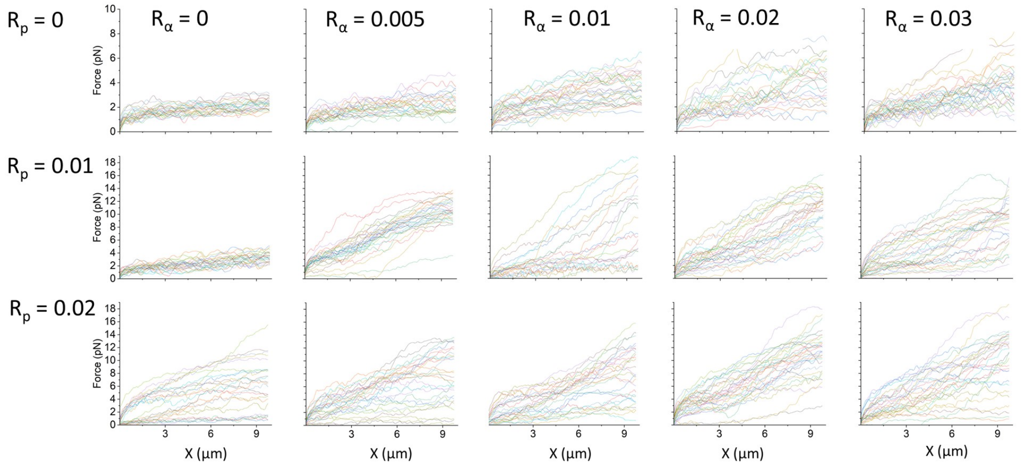

Figure S1. Individual force trials that are averaged over to compute the average force curves $F(x)$ shown in Fig 3A. Each trial is taken using a different microsphere probe in a different region of the sample chamber. Each panel corresponds to a different combination of R_α (columns) and R_p (rows) as indicated on the top and left of the panel grid.

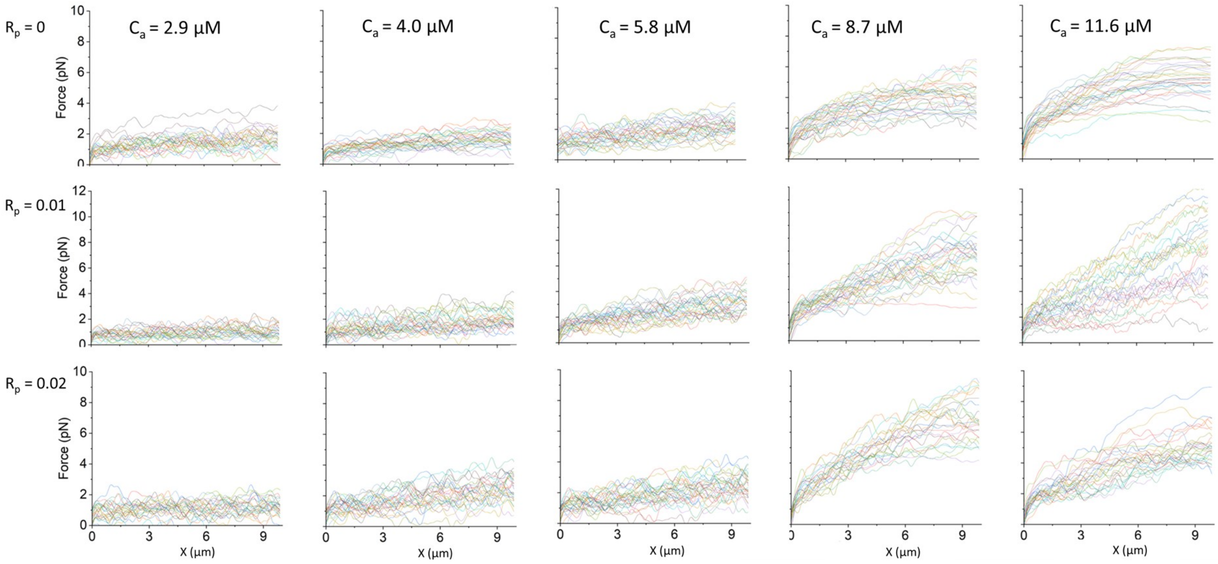

Figure S2. Individual force trials that are averaged over to compute the average force curves $F(x)$ shown in Fig 6 and SI Fig S3. Each trial is taken using a different microsphere probe in a different region of the sample chamber. Each panel corresponds to a different combination of actin concentration c_a (columns) and [phalloidin]:c_a ratio R_p (rows) as indicated on the top and left of the panel grid.

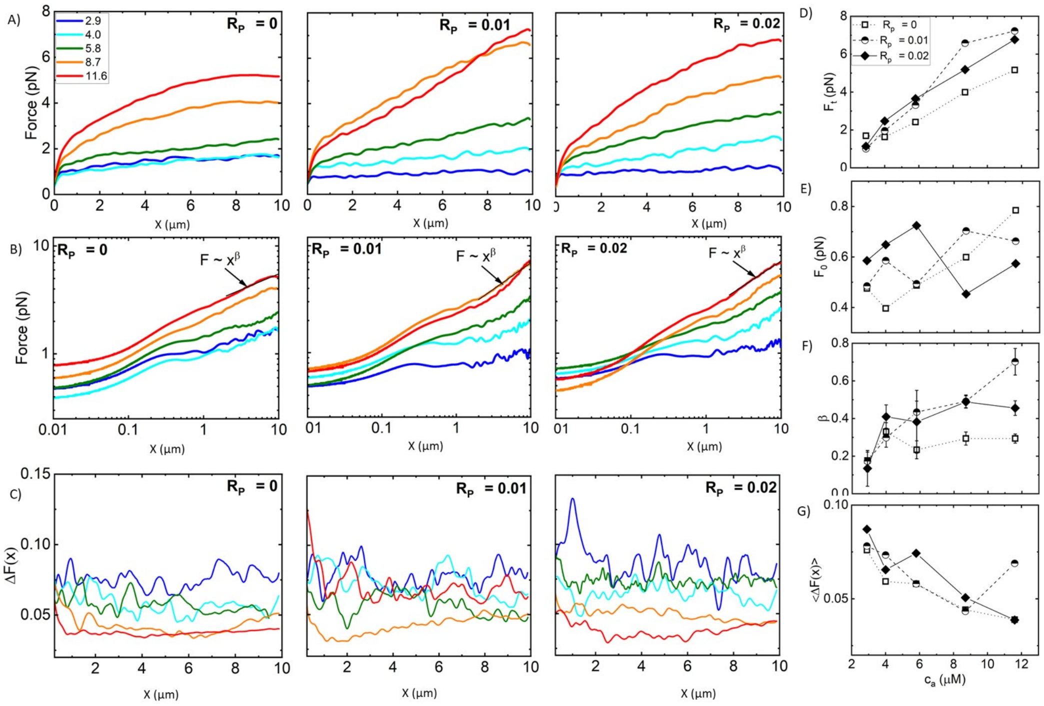

Figure S3. Nonlinear force response of actin networks of varying phalloidin and actin concentrations. (A) Average force $F(x)$ versus stage position x measured for actin networks subject to nonlinear straining. Different curves in each panel correspond to actin concentration c_a = 0 - 0.03, color-coded according to the legend, and different panels display data for phalloidin:actin molar ratios of R_p = 0 (left), R_p = 0.01 (middle), and R_p = 0.02 (right). (B) Data shown in (A) plotted on a log-log scale to highlight the trends seen for the initial force $F(x = 0) = F_0$ and power-law scaling of $F(x)$ near the end of the strain ($x \approx 1$). Fitting the large strain data to a power-law F(x)$\sim$ x^{β} yields the scaling exponent β. (C) Fractional spread in force $\Delta F(x)$ for each position x and each condition, determined by computing the standard error across 30 individual trials and normalizing by the average value plotted in A: $\Delta F(x) = SEF(x)/\langle F(x) \rangle$. (D-G) Metrics computed from the data shown in A-C as a function of c_a for R_p=0 (open squares, dotted connecting lines), R_p=0.01 (half-filled circles, dashed connecting lines), and R_p=0.02 (solid diamonds, solid connecting lines): (D) Terminal force reached at the end of the strain F_t; (E) Initial force measured at the beginning of the strain F_0; (F) Power-law scaling exponent β; (G) $\Delta F(x)$ averaged over the strain position x, resulting in a single value for each curve shown in (C) with error bars denoting the standard error across x values.

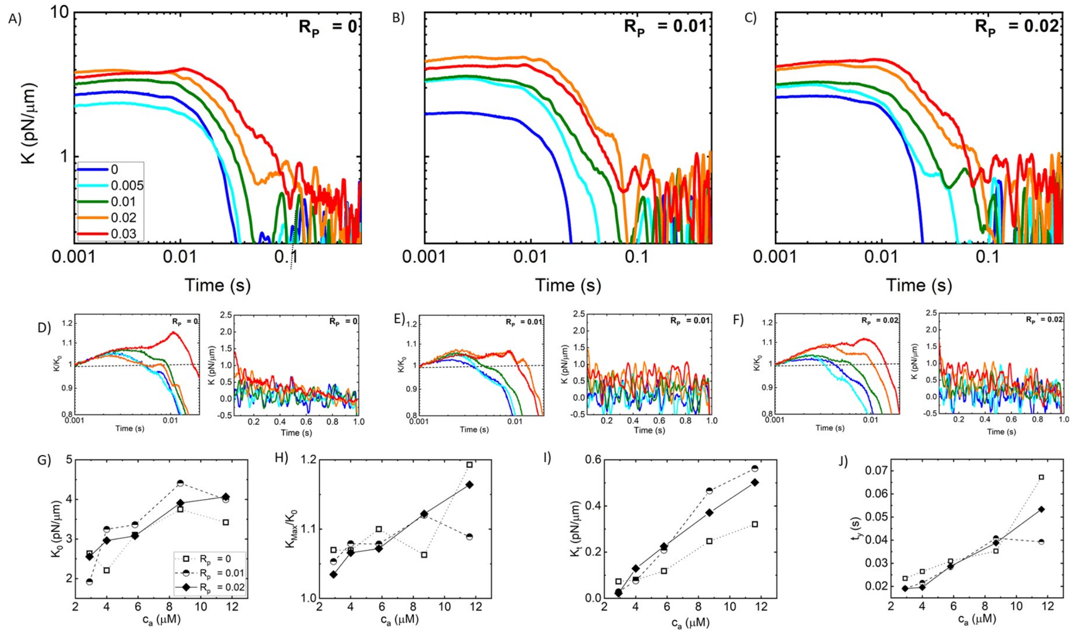

Figure S4. Effective differential modulus $K(t) = dF(x,t)/dx$ **as a function of time during strain** $t = v/x$**, computed from the data shown in SI Fig. S3A.** (A-C) The color-coded curves in each panel are for c_a values indicated in the legend and the different panels show data for (A) R_p = 0, (B) R_p = 0.01, and (C) R_p = 0.02. (D-F) $K(t)$ data shown in (A-C) normalized by the corresponding initial value K_0 are shown in the left panels. The horizontal dashed line at $K(t)/K_0$=1 guides the eye to show stress stiffening ($K(t)/K_0 > 1$) or softening ($K(t)/K_0 < 1$). Right panels show zoom-ins of $K(t)$ near the end of the strain where $K(t)$ is approximately constant. (G-J) Metrics computed from the data shown in A-C as a function of c_a for R_p=0 (open squares, dotted connecting lines), R_p=0.01 (half-filled circles, dashed connecting lines), and R_p=0.02 (solid diamonds, solid connecting lines): (G) Initial differential modulus K_0, (H) Degree of stress-stiffening, quantified as K_{max}/K_0, (I) Terminal stiffness K_t, and (J) yielding time t_y, defined as the time at which $K(t) = K_0/e$

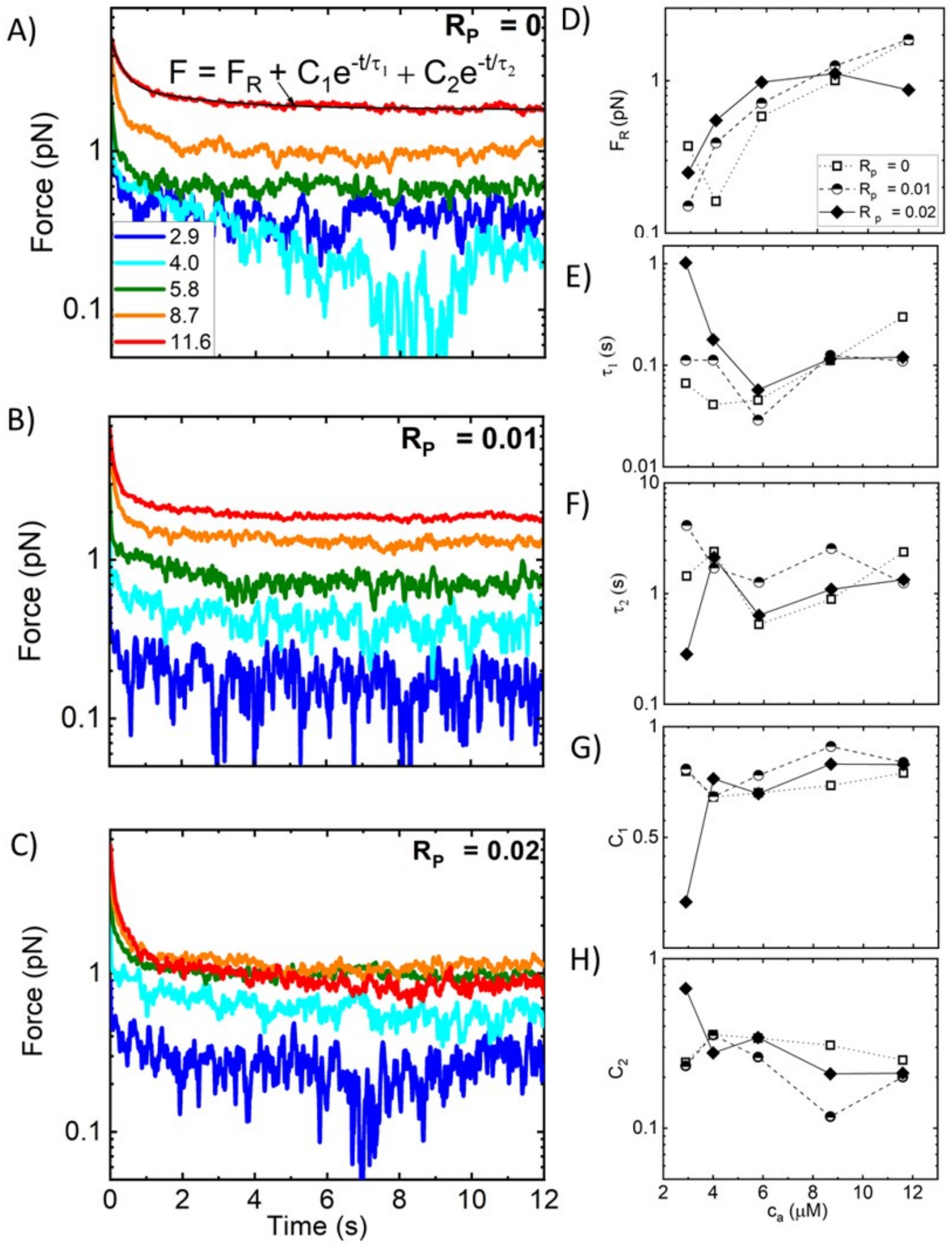

Figure S5. Dependence of nonlinear force relaxation on R_p and c_a. (A-C) Time-dependent relaxation of force $F(t)$ following strain for actin networks with varying c_a values, color-coded according to the legend, and R_p values of (A) 0, (B) 0.01, and (C) 0.02. All $F(t)$ curves are well described by a sum of two exponential decays with a long-time residual F_R: $F(t) = F_R + C_1 \exp(-t/\tau_1) + C_2 \exp(-t/\tau_2)$ as indicated by the representative fit (solid black line) shown in (A). (D-H) The residual force F_R (D), decay times τ_1 (E) and τ_2 (F), and corresponding fractional coefficients $c_1 = C_1/(C_1 + C_2)$ (G) and $c_2 = C_2/(C_1 + C_2)$ (H), are determined from the fits and plotted as functions of c_a for R_p=0 (open squares, dotted connecting lines), R_p=0.01 (half-filled circles, dashed connecting lines), and R_p=0.02 (solid diamonds, solid connecting lines).

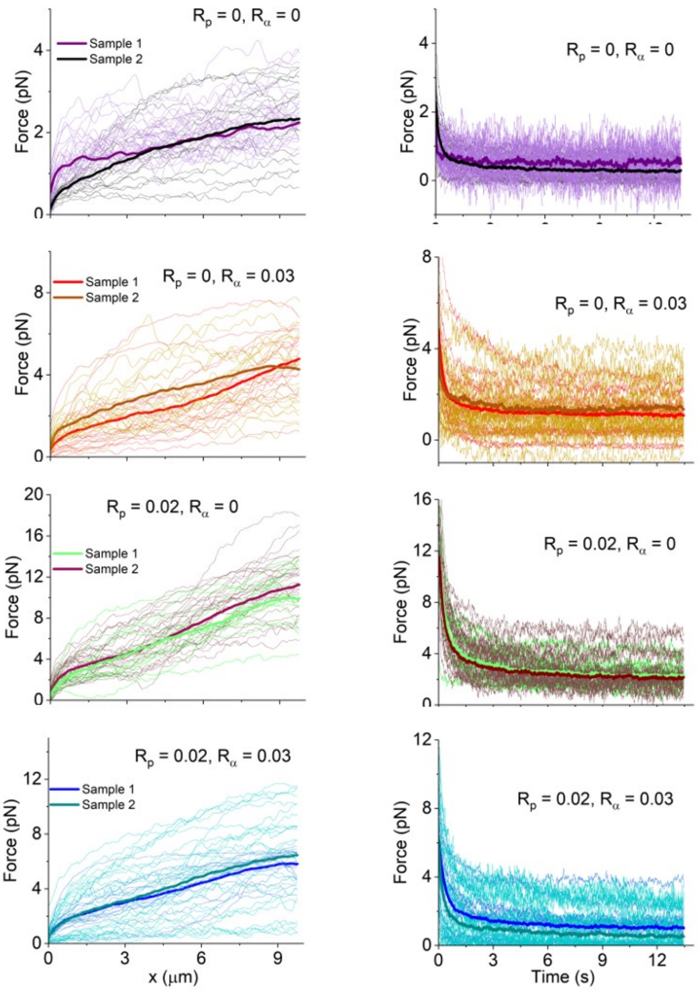

Figure S6. Comparison of force data measured for two independent samples for actin networks with four different (R_p, R_α) combinations: (0,0) (purple), (0,0.03) (orange), (0.02,0) (red/green), and (0.02, 0.03) (blue). Thin lines are individual trials and thick lines are the corresponding averages for the data shown in the paper (Sample 1) and data measured for a replicate sample for each (R_p, R_α) combination (Sample 2). As shown the strain-induced force (left) and relaxation (right) curves for both replicates are statistically indistinguishable, demonstrating the reproducibility and validity of our results

2. Supplemental Tables

Table 1. Table S1: Fit parameters and R^2 values determined from fitting the force relaxation curves shown in Fig 5 to the function $F(t) = F_R + C_1 \exp(-t/\tau_1) + C_2 \exp(-t/\tau_2)$.

Quantity	R_α	0	0.005	0.01	0.02	0.03
F_R	$R_p = 0$	0.52 ± 0.02	0.41 ± 0.08	0.98 ± 0.10	0.80 ± 0.12	1.36 ± 0.05
	$R_p = 0.01$	0.71 ± 0.02	1.01 ± 0.08	1.41 ± 0.10	1.58 ± 0.12	3.41 ± 0.05
	$R_p = 0.02$	0.52 ± 0.12	1.02 ± 0.04	1.32 ± 0.11	0.70 ± 0.13	2.11 ± 0.12
τ_1	$R_p = 0$	0.03 ± 0.01	0.07 ± 0.03	0.06 ± 0.02	0.12 ± 0.07	0.09 ± 0.03
	$R_p = 0.01$	0.03 ± 0.01	0.23 ± 0.03	0.34 ± 0.02	0.35 ± 0.07	0.36 ± 0.03
	$R_p = 0.02$	0.24 ± 0.02	0.24 ± 0.02	0.22 ± 0.01	0.19 ± 0.02	0.29 ± 0.02
τ_2	$R_p = 0$	1.34 ± 0.27	2.92 ± 0.35	0.64 ± 0.15	3.26 ± 0.26	1.51 ± 0.12
	$R_p = 0.01$	1.29 ± 0.11	1.87 ± 0.17	2.60 ± 0.19	2.88 ± 0.29	2.33 ± 0.40
	$R_p = 0.02$	3.99 ± 0.46	1.52 ± 0.80	1.22 ± 0.12	1.37 ± 0.20	2.86 ± 0.15
c_1	$R_p = 0$	0.80 ± 0.05	0.76 ± 0.02	0.66 ± 0.09	0.74 ± 0.11	0.66 ± 0.05
	$R_p = 0.01$	0.74 ± 0.09	0.82 ± 0.99	0.80 ± 0.09	0.86 ± 0.05	0.80 ± 0.04
	$R_p = 0.02$	0.82 ± 0.04	0.87 ± 0.06	0.79 ± 0.04	0.85 ± 0.03	0.79 ± 0.03
c_2	$R_p = 0$	0.20 ± 0.05	0.24 ± 0.06	0.34 ± 0.09	0.26 ± 0.11	0.34 ± 0.05
	$R_p = 0.01$	0.26 ± 0.09	0.18 ± 0.99	0.20 ± 0.09	0.14 ± 0.05	0.20 ± 0.04
	$R_p = 0.02$	0.18 ± 0.04	0.13 ± 0.06	0.21 ± 0.04	0.15 ± 0.03	0.21 ± 0.03
R^2	$R_p = 0$	0.65	0.83	0.89	0.92	0.92
	$R_p = 0.01$	0.78	0.99	0.99	0.99	0.99
	$R_p = 0.02$	0.99	0.99	0.99	0.99	0.99
